# Comparisons of Underlying Mechanisms, Clinical Efficacy and Safety Between Anti-PD-1 and Anti-PD-L1 Immunotherapy: The State-of-the-Art Review and Future Perspectives

**DOI:** 10.3389/fphar.2021.714483

**Published:** 2021-07-07

**Authors:** Yating Zhao, Liu Liu, Liang Weng

**Affiliations:** ^1^ Institute of Pharmaceutical Science, King’s College London, London, United Kingdom; ^2^ Clinical Pharmacology, BeiGene Ltd., Shanghai, China; ^3^ Ruikang Hospital Affiliated to Guangxi University of Chinese Medicine, Nanning, China; ^4^ Key Laboratory of Molecular Radiation Oncology, Changsha, China; ^5^ Xiangya Cancer Center, Xiangya Hospital, Central South University, Changsha, China; ^6^ Xiangya Lung Cancer Center, Xiangya Hospital, Central South University, Changsha, China

**Keywords:** immunotherapy, PD-1/PD-L1, nivolumab, pembrolizumab, tislelizumab, atezolizumab, efficacy, safety

## Abstract

Over the past decade, diverse PD-1/PD-L1 blockades have demonstrated significant clinical benefit in across a wide range of tumor and cancer types. With the increasing number of PD-1/PD-L1 blockades available in the market, differences between the clinical performance of each of them started to be reported. Here, we provide a comprehensive historical and biological perspective regarding the underlying mechanism and clinical performance of PD-1/PD-L1 blockades, with an emphasis on the comparisons of their clinical efficacy and safety. The real-world evidence indicated that PD-1 blockade may be more effective than the PD-L1, though no significant differences were found as regards to their safety profiles. Future head-to-head studies are warranted for direct comparison between them. Finally, we summarize the yet to be elucidated questions and future promise of anti-PD-1/PD-L1 immunotherapy, including a need to explore novel biomarkers, novel combinatorial strategies, and their clinical use on chronic infection.

## Introduction

Programmed cell death protein 1 (PD-1), also known as CD279, is a surface co-inhibitory protein that belongs to the immunoglobulin superfamily and is encoded by the PDCD1 gene in human. It was originally discovered being expressed in activated T lymphocytes (Alsaab et al., 2017). When PD-1 binds to its ligands, known as PD-L1 (B7-H1) and PD-L2 (B7-DC), it will trigger a dual mechanism of stimulating apoptosis in PD-1 expressed T-cells while simultaneously reducing apoptosis in regulatory T-cells, resulting in down-regulation of the immune system (McDermott and Atkins, 2013). The down-regulating immune response can protect healthy tissues from damage induced by excessive inflammation in the physiological environment, while in the tumor microenvironment, the significant over-expression of PD-1/PD-L1 protects tumor cells from apoptosis (Mahoney et al., 2015).

The understanding of PD-1/PD-L1 pathway provided evidence supporting the development of antibodies that inhibit the pathway, so called PD-1/PD-L1 blockades. Over the past few years, many PD-1/PD-L1 blockades have made a remarkable journey from the bench to the bedside and have led to significant clinical benefits to antitumor therapies (Makuku et al., 2021). With the increasing approved use of anti-PD-1/PD-L1 therapy in tumor immunotherapy, disparities between their clinical performance have attracted widespread attention by clinicians (Duan et al., 2020). Understanding the similarities and differences between PD-1 and PD-L1 blockade is needed to contribute ultimate benefits to patients with cancer.

In this review, we firstly introduce the PD-1/PD-L1 signaling pathway in normal immune function and in tumor microenvironment. The clinical use, efficacy and safety of marketing PD-1/PD-L1 blockades are then summarized, with the focus on their comparative clinical outcomes reported by several recent meta-analyses. Finally, we discuss future perspectives of PD-1/PD-L1 blockades in not only tumor/cancer immunotherapy but anti-chronic infections.

## PD-1/PD-L1 Signaling Pathway

**FIGURE 1:**
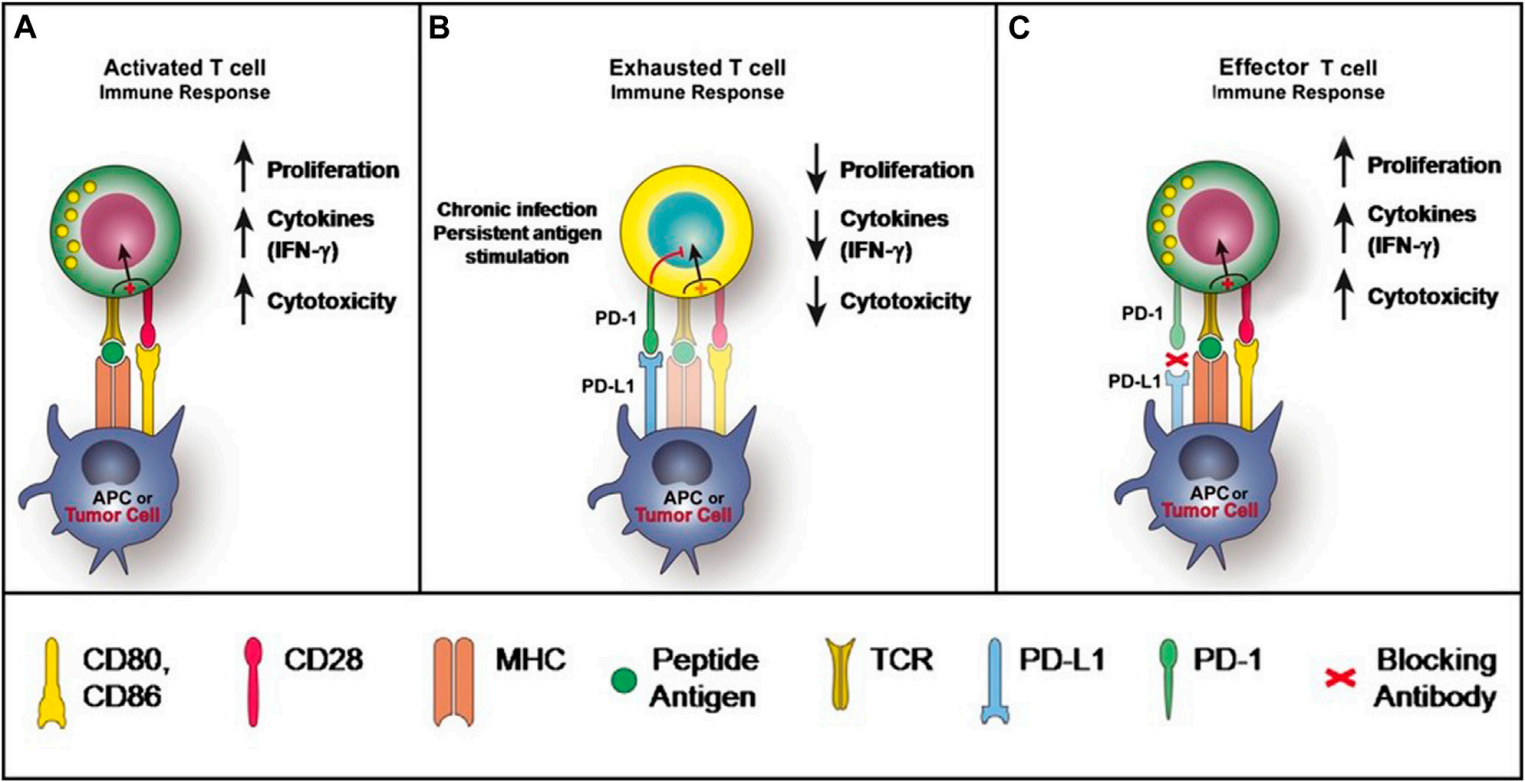
PD-1 and PD-L1 Signaling Pathway McDermott and Atkins (2013).

PD-1 is expressed on activated T cells, B cells, monocytes, and natural killer T cells, including cluster of differentiation (CD)8 + cytotoxic T lymphocytes and CD4^+^ T-helper lymphocytes (McDermott and Atkins, 2013). The two known ligands of PD-1: PD-L1 and PD-L2, both of which are expressed by antigen-presenting cells (APCs) and other immune cells, and can also be expressed on nonimmune cells, including tumor cells (Keir et al., 2008; Dong et al., 1999). PD-L1 is thought to be the principal mediator of PD-1-dependent immunosuppression (Brahmer et al., 2010). When a T cell recognizes the antigen expressed by the major histocompatibility complex (MHC) on the target cell, inflammatory cytokines are produced, initiating the inflammatory process. Upon T cell activation, PD-1 expression is induced. After PD-1 binding with PD-L1, the immunoreceptor tyrosine-based switch motif (ITSM) of PD-1 is phosphorylated to activate intracellular pathways to exert immunosuppression activities: on one hand, the TCR activation signals ZAP70 and CD3δ are immediately dephosphorylated, leading to downstream PI3K/Akt pathway repression and then decreases the cell apoptosis-related gene Bcl-xl and promotes T cell apoptosis (Hofmeyer et al., 2011); on the other hand, Ras/MEK/ERK pathway is inhibited to repress T cell proliferation (Patsoukis et al., 2012). Alternatively, PD-1/PD-L1 pathway impairs the cytokine secretion released by T cells (Hofmeyer et al., 2011). Altogether, PD-L1 signals through T-cell PD-1 to negatively regulates the T-cell receptor and attenuates T-cell proliferation and functional activities, leading to T-cell exhaustion (Figure 1). Nevertheless, the inhibitory mechanism of PD-1/PD-L1 pathway differs between T and B cells. In B cells, following PD-1 activation, BCR pathway molecules, such as Igα/β and SγK, are dephosphorylated via SHP-2 being recruited to PD-1, therefore inhibiting PI3K, ERK and PLCγ2 pathway, leading to Ca2+ disorder and B cell growth stagnation (Okazaki et al., 2001; Nicholas et al., 2013). Physiologically, the PD-1/PD-L1 signaling pathway control the degree of inflammation at locations expressing the antigen, minimizing damage to healthy tissue (Mahoney et al., 2015).

However, this protective mechanism triggered by PD-1/PD-L1 is perverted in certain tumors. In the tumor microenvironment, the expression of PD-L1 can be markedly upregulated on tumor cells in the presence of interferon-gamma (IFN-γ), while the expression of PD-1 is significantly lifted on tumor-infiltrating lymphocytes. The engagement of PD-L1 with PD-1 of T cells leads to T-cell dysfunction, exhaustion, neutralization, and interleukin-10 (IL-10) production in a tumor mass (Sun et al., 2015). As a result, T cells unable to destroy the tumor, further enabling tumor cell evasion of immune destruction (Alsaab et al., 2017) (Figure 1). The function of PD-1 in B-cells have also become apparent for tumor immunosuppression (Thibult et al., 2013). Furthermore, it has been reported that PD-L1 can increase the expression of Foxp3 (the transcription factor controlling regulatory T-cell [Treg] development) and convert naive CD4^+^ T cells to Tregs through the downregulation of Akt, mTOR and ERK2 and the simultaneous upregulation of PTEN (Francisco et al., 2010). The expansion of Tregs further execute their immunosuppressive abilities in the tumor microenvironment through maintaining the expression of PD-1 on its surface (Francisco et al., 2010).

It is found that a variety of tumors, including renal cell cancer (RCC), melanoma (MEL), as well as stomach, breast, ovarian, pancreatic, and lung cancers, express PD-L1, potentially contributing to immune suppression and evasion (Zou and Chen, 2008). Consequently, therapies that inhibit the PD-1/PD-L1 pathway can restore the antitumor immune responses and be particularly beneficial to patients with PD-L+ tumors, which has been proved in many clinical studies of checkpoint inhibitors (Duan et al., 2020). However, it has been noted that not all tumor PD-L1 expression confers a worse prognosis (Taube et al., 2012), and further work on this question is ongoing.

## Clinical Use, Efficacy and Safety

Numbers of PD-1 and PD-L1 inhibitors were developed and widely used in a wide ranges of tumor types. In 2014, the humanized anti-PD-1 monoclonal antibody nivolumab became the first FDA-approved anti-PD-1 regimen for unresectable or metastatic melanoma (Weber et al., 2015; Robert et al., 2015a). In the same year, pembrolizumab was also approved for unresectable or metastatic melanoma (Robert et al., 2014). In the upcoming years, several novel monoclonal antibodies against PD-1, toripalimab, sintilimab, camrelizumab, tislelizumab and cemiplimab received approval for marketing consecutively. Further clinical trials succeeded and indications expanded to non-small cell lung cancer (NSCLC), renal cell cancer (RCC), urothelial carcinoma (UC), squamous cell carcinoma of the head and neck (HNSCC) and hepatocellular carcinoma (HCC) (Table 1). In 2016, first anti-PD-L1 antibody atezolizumab was approved for locally advanced or metastatic UC based on an improved objective response rate (ORR) (Rosenberg et al., 2016). Following that, durvalumab and avelumab, two specific antibodies against PD-L1 were approved to enter the market. Similar to anti-PD-1 antibodies, anti-PD-L1 antibodies have been effective in some difficult-to-treat cancer (Table 2).

**TABLE 1:** Clinical use of anti-PD-1 antibodies.

Drugs	Indication	Regimens	
		Monotherapy	Combination therapy
Nivolumab			
	Melanoma, NSCLC, SCLC, renal cell carcinoma, Hodgkin’s lymphoma, HNSCC, colorectal cancer, HCC, urothelial cancer	Nivolumab 3 mg/kg q2w	
	Esophageal squamous cell cancer	Nivolumab 240 mg q2w	
	Melanoma without BRAF mutation for 1st line		Nivolumab 1 mg/kg + ipilimumab 3 mg/kg q3w, 4 doses, then nivolumab 3 mg/kg q2w
	NSCLC (PD-L1 ≥ 1%) for 1st line		Nivolumab 360 mg q3w + ipilimumab 1 mg/kg q6w
	NSCLC for 1st line		Nivolumab 360 mg q3w + ipilimumab 1 mg/kg q6w + platinum doublet chemotherapy q3w, 2 cycles
	Renal cell carcinoma for 1st line, colorectal cancer		Nivolumab 3 mg/kg q3w + ipilimumab 1 mg/kg q3w, 4 doses, then nivolumab 3 mg/kg q2w
	Pleural mesothelioma		Nivolumab 3 mg/kg q2w + ipilimumab 1 mg/kg q6w
Pembrolizumab			
	Melanoma, NSCLC (PD-L1 ≥ 1%), NSCLC (PD-L1 ≥ 50%), SCLC, HNSCC, Hodgkin's lymphoma, primary mediastinal B-cell lymphoma, urothelial cancer, colorectal cancer, gastric cancer, esophageal cancer, cervical cancer, HCC, Merkel cell carcinoma, cutaneous squamous cell carcinoma	Pembrolizumab 200 mg q3w	
	NSCLC without EGFR or ALK mutation		Pembrolizumab 200 mg q3w + (pemetrexed + platinum-based drug) q3w, 4 cycles, then pembrolizumab + pemetrexed maintenance
	HNSCC		Pembrolizumab + 5-fluorouracil + platinum
	Renal cell carcinoma		Pembrolizumab 200 mg q3w + axitinib 5 mg bid
Toripalimab			
	Melanoma	Toripalimab 3 mg/kg q2w	
Sintilimab			
	Hodgkin lymphoma	Sintilimab 200 mg q3w	—
Camrelizumab			
	Hodgkin lymphoma, esophageal cancer	Camrelizumab 200 mg q2w	
	HCC	Camrelizumab 3 mg/kg q3w	
	NSQ NSCLC		Camrelizumab 200 mg q3w + (carboplatin + pemetrexed) q3w 4–6 cycles, then pemetrexed with/without camrelizumab maintenance
Tislelizumab			
	Hodgkin lymphoma, urothelial cancer	Tislelizumab 200 mg q3w	
Cemiplimab			
	Cutaneous squamous cell carcinoma	Cemiplimab 3 mg/kg q2w	

**TABLE 2:** Clinical use of anti-PD-L1 antibodies.

Drugs	Indication	Regimens	
		Monotherapy	Combination therapy
Atezolizumab			
	Urothelial cancer, NSCLC	Atezolizumab 1200 mg q3w	
	NSCLC		Atezolizumab + carboplatin + paclitaxel
	SCLC		Atezolizumab + carboplatin + etoposide
	Breast cancer		Atezolizumab + nab-paclitaxel
	HCC		Atezolizumab + bevacizumab
	Melanoma		Atezolizumab + cobimetinib + vemurafenib
Durvalumab			
	Urothelial cancer, stage III NSCLC	Durvalumab 10 mg/kg q2w	
	ES-SCLC		Durvalumab 1500 mg + etoposide + carboplatin or cisplatin
Avelumab			
	Merkel cell carcinoma, urothelial cancer	Avelumab 10 mg/kg q2w	
	Urothelial cancer		Gemcitabine + cisplatin or carboplatin 4 cycles, then maintenance avelumab
	RCC		Avelumab 10 mg/kg q2w + axitinib 5 mg bid

### Nivolumab

The clinical development of nivolumab was initiated in 2010. In a phase 1 trial, nivolumab (MDX-1106) exhibited an evidence of antitumor activity and was well tolerated (Brahmer et al., 2010). Further clinical trial that assessed the activity and safety of nivolumab (previous known as BMS-936558) demonstrated anti-PD-1 antibody produced antitumor responses in melanoma, NSCLC and RCC (Topalian et al., 2012). In the phase 3 trials, nivolumab showed a higher rate of objective response than chemotherapy regimens in patients with advanced melanoma who had disease progression after ipilimumab or a BRAF inhibitor (Weber et al., 2015) and a better overall survival (OS) of 72.9% at 1 year compared with 42.1% in the dacarbazine group as a first-line treatment (Robert et al., 2015a). Moreover, clinical trial of combination therapy demonstrated nivolumab combined with ipilimumab had a longer free-progression survival (PFS) than nivolumab alone or ipilimumab alone (11.5, 6.9 and 2.9 months respectively) in advanced melanoma (Larkin et al., 2015). In 2014, FDA granted nivolumab approval for treatment of unresectable or metastatic melanoma. And several months later, nivolumab was approved for NSCLC with progression after chemotherapy. In the phase 3 trials of NSCLC, nivolumab provided a 3.2 months increase of OS in squamous (SQ) NSCLC and 2.8 months increase in nonsquamous (NSQ) NSCLC compared with docetaxel (Borghaei et al., 2015; Brahmer et al., 2015). Regardless of PD-L1 expression, nivolumab plus ipilimumab with or without chemotherapy provided OS benefit compared with chemotherapy alone in untreated metastatic NSCLC (Hellmann et al., 2019; Paz-Ares et al., 2021). As neoadjuvant therapy in NSCLC, nivolumab plus chemotherapy demonstrated superior efficacy with a pCR of 24% comparing to chemotherapy alone (Janjigian et al., 2018) and it will be a new way of treating resectable NSCLC. Based on successful clinical trials, the use of nivolumab has been expanded to small-cell lung cancer (SCLC) (Ready et al., 2019), RCC (Motzer et al., 2015; Motzer et al., 2018), Hodgkin lymphoma (Younes et al., 2016), HNSCC (Ferris et al., 2016), colorectal cancer (Overman et al., 2017; Overman et al., 2018), HCC (El-Khoueiry et al., 2017), esophageal squamous cell cancer (ESCC) (Kato et al., 2019), UC (Sharma et al., 2017) and pleural mesothelioma (Scherpereel et al., 2019).

### Pembrolizumab

The clinical development of pembrolizumab (previously known as lambrolizumab or MK-3475) started with advanced melanoma (Hamid et al., 2013). In the phase 1 trial, pembrolizumab showed an ORR of 26% at both low-dosage (2 mg/kg q3w) and high-dosage (10 mg/kg q3w) in patients with advanced melanoma after treatment of ipilimumab (Robert et al., 2015b). In head-to-head comparison with ipilimumab it increased ORR and prolonged PFS and OS in patients with advanced melanoma (Ribas et al., 2015). Further phase 1b trials demonstrated that pembrolizumab exhibited antitumor activity in advanced triple-negative breast cancer (TNBC) (Nanda et al., 2016), advanced gastric cancer (GC) (Muro et al., 2016), HNSCC (Seiwert et al., 2016) and UC (Plimack et al., 2017). In 2014, FDA approved pembrolizumab for the second-line treatment of melanoma. Pembrolizumab was also approved for the treatment in patients with PD-L1-expressing NSCLC (Herbst et al., 2016; Reck et al., 2016) and increased expression of PD-L1 on tumor cells was associated with improved efficacy (Garon et al., 2015). Moreover, the addition of pembrolizumab to standard chemotherapy resulted in a significantly longer OS than chemotherapy alone as first-line therapy, which supported to be a standard treatment for metastatic NSQ NSCLC (Gandhi et al., 2018). Similar to nivolumab, the indications of pembrolizumab have been extended to SCLC (Gadgeel et al., 2018), HNSCC (Cohen et al., 2019), Hodgkin's lymphoma (Chen et al., 2017), primary mediastinal B-cell lymphoma (PMBCL) (Armand et al., 2019), UC (Balar et al., 2017; Bellmunt et al., 2017), colorectal cancer (Andre et al., 2020; Le et al., 2020), GC (Fuchs et al., 2018), esophageal cancer (Kojima et al., 2020), cervical cancer (Chung et al., 2019), HCC (Finn et al., 2020a), Merkel cell carcinoma (Nghiem et al., 2019), cutaneous squamous cell carcinoma (cSCC) (Grob et al., 2020) and RCC (Rini et al., 2019). Moreover, pembrolizumab became the first drug to be approved for advanced MSI-H/dMMR-positive solid tumors based on a tumor-specific biomarker instead of the cancer location (Boyiadzis et al., 2018).

### Toripalimab

The humanized IgG4 anti-PD-1 mAb toripalimab (previous known as JS001) is developed by Shanghai Junshi Bioscience Co., Ltd. in China. The phase 1 trial demonstrated antitumor activity in UC, RCC and melanoma, especially in previous underexplored acral and mucosal melanoma subtypes (Tang et al., 2019). Further clinical trial revealed toripalimab provided an OS of 22.2 months in patients with acral and mucosal melanoma (Tang et al., 2020). Based on the positive efficacy of this trial, toripalimab received conditional approval for second-line treatment of metastatic melanoma in China. Plenty of clinical trials are ongoing, including monotherapy for treatment of advanced GC (Wang et al., 2019), neuroendocrine neoplasms (Lu et al., 2020) and NSCLC (Wang et al., 2020a), as well as combination therapy for mucosal melanoma (Sheng et al., 2019) and ESCC (Xing et al., 2020).

### Sintilimab

Sintilimab is a fully humanized mAb against PD-1 receptor which is co-developed by Innovent Biologics and Eli Lilly Company. In 2018, sintilimab was approved for the treatment of relapsed or refractory classical Hodgkin lymphoma after two lines or more chemotherapy because it provided a high ORR of 80.4% (Shi et al., 2019). The phase 3 trial of sintilimab provided a longer 5.3 months of OS than docetaxel in patients with NSCLC whose disease progressed after chemotherapy (Yang et al., 2020). Besides of monotherapy, sintilimab combined with either chemotherapy or anlotinib as the first-line treatment demonstrated encouraging antitumor activities (Chu et al., 2021; Jiang et al., 2021) and combined with anlotinib, it showed a longer PFS of 15 months representing a novel chemotherapy-free regimen of NSCLC (Zhang et al., 2021). The addition of sintilimab to chemotherapy also revealed promising efficacy and manageable safety in untreated gastric/gastroesophageal junction (GEJ) adenocarcinoma (Jiang et al., 2020).

### Camrelizumab

Camrelizumab, previous known as SHR-1210, is developed by Jiangsu Hengrui Medicine Co., Ltd. The phase 1 trial of camrelizumab exhibited promising antitumor activity with two complete responses (in GC and bladder carcinoma) (Mo et al., 2018). Camrelizumab showed an ORR of 76.0% and controllable safety in patients with classical Hodgkin lymphoma after at least two lines of treatment, leading to the approval for treatment of classical Hodgkin lymphoma in China (Song et al., 2019b). In combination with chemotherapy, it provided a 4-months increased in PFS compared with chemotherapy alone in untreated patients with NSQ NSCLC (Zhou et al., 2021). At present, the clinical use of camrelizumab has been expanded to HCC (Qin et al., 2020) and esophageal cancer (Huang et al., 2020). It is still being investigated for the treatment of B-cell lymphoma (Mei et al., 2020), gastric/GEJ carcinoma (Huang et al., 2019) and nasopharyngeal carcinoma (Fang et al., 2018).

### Tislelizumab

Tislelizumab which was developed by BeiGene has been investigated in solid tumors and hematological cancers since 2015. The phase 1/2 studies of tislelizumab demonstrated an acceptable safety and antitumor activity in patients with advanced solid tumors (Desai et al., 2020; Shen et al., 2020). In the treatment of classical Hodgkin lymphoma, tislelizumab was well tolerated with a high ORR of 87.1% (Song et al., 2019a). It also provided an OS of 9.8 months in patients with UC (Ye et al., 2021). Those results led to its approval for classical Hodgkin lymphoma and UC in China. Recent studies in patients with NSCLC progressed after chemotherapy, tislelizumab showed a 3.54 months increase of OS and a 1.51 months increase of PFS comparing to docetaxel. In addition to monotherapy, clinical trials of tislelizumab plus chemotherapy as first-line treatment are investigated in ESCC and gastric/GEJ adenocarcinoma (Xu et al., 2020) and lung cancer (Wang et al., 2020b) are ongoing.

### Cemiplimab

In 2018 cemiplimab became the first FDA-approved PD-1-targeted therapeutics for advanced cutaneous squamous cell carcinoma that no systemic therapy has been approved. Among patients with advanced cSCC, almost half of patients responded to cemiplimab (Migden et al., 2018; Migden et al., 2020). Further clinical trials demonstrated cemiplimab produced substantial antitumor activity at either weight-based dose (3 mg/kg q2w) or fixed-dose (350 mg q3w) (Rischin et al., 2020). In NSCLC with PD-L1 expression at least 50%, cemiplimab provided a longer PFS than platinum-doublet chemotherapy, although median OS has not reached with cemiplimab (Sezer et al., 2021).

### Atezolizumab

Atezolizumab (previously known as MPDL3280A) is the first-approved PD-L1 blockade for treatment of UC. An ORR of 15% was significantly improved compared with a historical control data (Rosenberg et al., 2016). However, additional clinical data indicated that atezolizumab could not provide survival benefit compared with chemotherapy in UC (Powles et al., 2018), while addition of atezolizumab to platinum-based chemotherapy prolonged PFS as first-line treatment (Galsky et al., 2020). Atezolizumab revealed promising antitumor activity in NSCLC. It provided 7.1 months longer in OS than platinum-based chemotherapy in PD-L1 high-expression patients with NSCLC (Herbst et al., 2020), resulting in its approval as first-line monotherapy for adults with metastatic NSCLC whose tumors are EGFR and ALK wild-type but have PD-L1 stained ≥50% of tumor cells or PD-L1 stained tumor-infiltrating immune cells covering ≥10% of the tumor area in 2020 (FDA). The OS was also improved compared with chemotherapy regardless of PD-L1 expression in previous treated NSCLC (Rittmeyer et al., 2017). Consequently, atezolizumab has also been approved for NSCLC regardless of PD-L1 expression either alone or in combination with chemotherapy. Indications of atezolizumab have been expanded to SCLC (Horn et al., 2018), TNBC (Schmid et al., 2018), HCC (Finn et al., 2020b) and melanoma (Gutzmer et al., 2020).

### Durvalumab

Durvalumab with or without tremelimumab demonstrated antitumor responses in multiple forms of solid tumors. At present, durvalumab is used for stage III NSCLC (Antonia et al., 2017), ES-SCLC (Paz-Ares et al., 2019) and UC (Powles et al., 2017). The phase 1/2 trial of advanced UC showed an ORR of 17.8% regardless of PD-L1 expression (Powles et al., 2017). Similar to atezolizumab, further study data has not demonstrated that durvalumab has survival benefit beyond chemotherapy in UC (Powles et al., 2020a). What’s more, durvalumab plus tremelimumab showed antitumor activity in NSCLC in a phase 1b study (Antonia et al., 2016). Later study data indicated durvalumab alone or combined with tremelimumab improved OS and PFS compared with standard of care as third-line or later treatment (Planchard et al., 2020). Numerous clinical trials are investigated in HNSCC (Ferris et al., 2020), TNBC (Loibl et al., 2019), HER2-positive breast cancer (Chia et al., 2019), gastric and GEJ adenocarcinoma (Kelly et al., 2020), lymphoma (Herrera et al., 2020) and pleural mesothelioma (Nowak et al., 2020).

### Avelumab

The phase 1 clinical trial that assessed the activity and safety of avelumab (MSB0010718C) demonstrated PD-L1 blockade produced antitumor responses in NSCLC (Gulley et al., 2017), UC (Apolo et al., 2017), breast cancer (Dirix et al., 2018), adrenocortical carcinoma (Le Tourneau et al., 2018), melanoma (Keilholz et al., 2019), mesothelioma (Hassan et al., 2019), ovarian cancer (Disis et al., 2019), RCC (Vaishampayan et al., 2019). Based on an ORR of 31.8% in patients with Merkel cell carcinoma in phase 2 study, avelumab became the first-approved anti-PD-L1 antibody for this rare and aggressive skin cancer (Kaufman et al., 2016). Avelumab has been approved for UC and RCC as well. The phase 1b trial of UC demonstrated avelumab provided an OS of 13.7 months and OS rate of 54.3% at 1 year (Apolo et al., 2017) and as maintenance therapy of first-line chemotherapy, OS was significantly longer than best supportive care (Powles et al., 2020b). In RCC, avelumab monotherapy showed clinical activity in both first-line and second-line treatment (Vaishampayan et al., 2019). Avelumab combined with axitinib provided a 5.4 months increase in PFS vs. sunitinib and was more effective than sunitinib among patients with untreated RCC (Motzer et al., 2019).

### Comparison of Efficacy Between PD-1 and PD-L1 Inhibitors

Immune checkpoint inhibitors targeting PD-1/PD-L1 pathway represent the new standard of care in wide spectrum of solid tumors and hematological cancers. While it remains controversial whether anti-PD-1 and anti-PD-L1 antibodies have comparative efficacy and safety in different forms of tumor.

Although in absence of head-to-head comparisons, some systematic reviews and meta-analyses have been done to access the difference between PD-1 and PD-L1 inhibitors through indirect comparisons. In patients with previous treated NSCLC, one meta-analysis data demonstrated nivolumab and pembrolizumab increased ORR compared with atezolizumab but no significant difference in OS and PFS was observed (Passiglia et al., 2018). Other reported the similar result that anti-PD-1 antibodies achieved better efficacy compared with anti-PD-L1 antibody as monotherapy in patients with pre-treated NSCLC (Almutairi et al., 2019). In combination with chemotherapy, pembrolizumab may have superior efficacy compared to atezolizumab in advanced squamous NSCLC as first-line treatment (Zhang et al., 2018). Besides of NSCLC, patients with HNSCC seemed to benefit more from anti-PD-1 therapy than from durvalumab based on an indirect analysis (Zhu et al., 2021). A meta-analysis based on mirror principle suggested patients obtained survival benefit from anti-PD-1 antibodies compared with anti-PD-L1 antibodies across different forms of tumor (NSCLC, GC, UC and RCC) in either monotherapy or combination therapy (Duan et al., 2020). However, in UC the clinical outcomes of PD-1 and PD-L1 inhibitors were similar when patients progressed after a platinum-based chemotherapy (Niglio et al., 2019). Others also suggested the efficacy was similar between anti-PD-1 and anti-PD-L1 across different tumor types (Weng et al., 2018). Few clinical data is available for inclusion, the lack of comparability and systematic bias might be potential limitations of some systematic reviews and meta-analyses.

Real-world evidence is necessary to complement trial evidence and crucial for helping clinicians tailor novel immunotherapy. After failure of anti-PD-1 antibodies, retreatment with atezolizumab revealed limited efficacy in most retreated patients in NSCLC, however it was no correlation between efficacy of prior anti-PD-1 treatment and efficacy of retreatment with atezolizumab (Furuya et al., 2021). And in real-world head-to-head comparison in metastatic melanoma for frontline therapy, efficacy of nivolumab was similar to that of pembrolizumab and no significant difference in OS was observed (Moser et al., 2020). Furthermore, a meta-analysis that enrolled 32 studies of daily clinical practice demonstrated anti-PD-1 and anti-PD-L1 immunotherapy provided survival benefits as sconed-line treatment of NSCLC, in which the median PFS and OS were 3.35 and 9.98 months, respectively (Mencoboni et al., 2021). Although most patients enrolled in this meta-analysis were treated with nivolumab, the efficacy in clinical practice is comparable to that in clinical trials. It might be difficult to determine a better treatment of either anti-PD-1 or anti-PD-L1. Clinical practice of choosing either drugs is based on patients and clinician preference as well as adverse events.

### Comparison of Safety and Tolerability Between PD-1 and PD-L1 Inhibitors

Anti-PD-1/PD-L1 have provoked a total paradigm shift in the treatment of oncological malignancies, thus a different pattern of toxicity has arisen in comparison with traditional chemotherapy agents. Main adverse events related to anti-PD-1/anti-PD-L1 agents are immune-related, with multiple organ and system being involved, such as hematology, cardiology, respiratory, ophthalmology, et al. (Baraibar et al., 2019). The immune-related adverse events are usually manageable, but toxicities may sometimes lead to treatment withdrawal, and fulminant and fatal events can also occur (Wang et al., 2019). Similarly, no head-to-head trial has been conducted to compare the difference of safety and tolerability between PD-1 and PD-L1. Until now, only two systematic review and meta-analyses reported the comparative incidence of immune-related adverse events of PD-1/PD-L1 via real-world data and indirect comparisons. Wang et al. (2019) conducted the meta-analysis of 112 trials involving 19,217 patients and reported toxicity-related fatality rates of 0.36% for anti–PD-1, and 0.38% for anti–PD-L1. Following that, Duan et al. (2020) found no significant difference in safety profiles between anti-PD-1 and anti-PD-L1 via the meta-analysis of 19 randomized clinical trials involving 11,379 patients.

Without a randomized, head-to-head trial of anti-PD-1 vs. anti-PD-L1 agents, no conclusive statements can be made regarding the comparative efficacy and safety of them. It appears, however, that targeting only PD-L1 may be less effective than targeting PD-1. One possible reason for the possibly superior efficacy of anti-PD-1 is that it can block signaling via both PD-L1 and PD-L2, while anti-PD-L1 only inhibit the binding the PD-1 to PD-L1. Another reason could be that anti-PD-L1 is overconsumed owing to extra PD-L1 expression following chemotherapy, leading to the inhibition of T-cell activation (Duan et al., 2020). In addition, the tumor burden, tumor growth kinetics, and tumor heterogeneity play important roles in drug resistance in cancer (Vasan et al., 2019), which has been proved by the inevitable emergence of drug resistance observed in many targeted cancer therapies (Lim et al., 2019). Therefore, the anti-PD-L1 targeting to the PD-L1 on tumor cells may be more easily resisted in comparison with anti-PD-1 targeting to the PD-1 on immune cells, which needs to be explored in future studies.

## Future Perspectives

### Identification of Novel Biomarkers

It has been noted that discovering novel predictive, diagnostic, and prognostic pharmacological biomarkers is beneficial to better clinical outcomes and fewer adverse effects for immunotherapy (Ribas et al., 2015). As an example, Higgs et al. (2018) proposed that T cell infiltration assessment or IFN-γ gene signature could be a promising predictive biomarker of PD-1/PD-L1 therapy. It has triggered the development of various assays to monitor T cell infiltration and detect novel biomarkers, such as PD-1/L1-targeted positron emission tomography-(PET-) based imaging biomarkers (Abousaway et al., 2021; Wei et al., 2018), single-cell sequencing technologies (Yu et al., 2016), Cytometry by Time-Of-Flight (CyTOF) (Kay et al., 2016), and genomic approaches (Hellmann et al., 2019), et al. Other potential biomarkers that have been recently found to be correlated to the clinical outcomes of PD-1/L1 include the gut microbiome (e.g. Akkermansia muciniphila), peripheral blood biomarkers (e.g. pretreatment neutrophil to lymphocyte ratio [NLR]), circulating microRNAs. However, their exact mechanisms are not clearly understood (Makuku et al., 2021).

### Combination Therapies

In 2018, Mahoney et al. divided tumor immune-cell infiltration into three types: 1) “immune-desert” or noninflamed, 2) “hot” or inflamed, and 3) immune-excluded. Following that, several agents have been reported to be effective to turn “cold” tumors to “hot” T cell infiltrative tumors, improving the effectiveness of anti-PD-1/PD-L1 immunotherapy. For example, anti-cytotoxic T-lymphocyte-associated protein 4 (CTLA-4) agents improve T cell infiltration into the tumor microenvironment, which provides an opportunity for PD-1 blockade agents to work more efficiently, and their synergic combination has been proved to be more efficacious and safer compared with PD-1 blockade or anti-CTLA-4 monotherapy (Wu et al., 2019). The research on other effective approaches to induce effector T cell infiltration into the tumors and improve the therapeutic efficacy of PD-1 is gaining more and more attentions, such as amalgamating PD-1 blockade with oncolytic viruses, cancer vaccines, and local ablation, et al. (Makuku et al., 2021).

Combination of PD-1/PD-L1 blockade with new blockades that inhibit a wider spectrum of inhibitory receptors is the current focus of immunotherapeutic research. In addition to PD-1 and CTLA-4, the inhibitory receptors leading to the failure of cancer elimination known until now include T-cell immunoglobulin mucin-3 (TIM-3), and lymphocyte activation gene 3 (LAG-3), T cell immunoglobulin and ITIM domain (TIGIT), and Band T-lymphocyte-associated protein (BTLA) receptors associated with T cell exhaustion as well as V-domain immunoglobulin suppressor of T cell activation (VISTA). Many clinical trials investigating their combinatorial strategies are ongoing, with the aim to yield better outcomes for cancer patients (Song et al., 2017).

### Treatment of Chronic Infections

As mentioned earlier, PD-1 and PD-L1 also play a key role in the failure to eliminate pathogens during chronic infections. Upregulation of PD-1 has been reported on T cells that are specific to tuberculosis (TB) (Jurado et al., 2008), human immunodeficiency virus (HIV) (Trautmann et al., 2006; Paris et al., 2015), hepatitis B virus (HBV) (Boni et al., 2007), and human T-cell leukemia virus type 1 (HTLV-1) (Kozako et al., 2009; Kozako et al., 2011). Also, the upregulation of PD-L1 has been reported on human gastric epithelial cells during a *Helicobacter pylori* infection (Das et al., 2006). It indicates that the PD-1 pathway also appears to result in insufficient clearance during specific bacterial infection. Therefore, anti-PD-1/PD-L1 therapy holds promise as adjunctive therapy for chronic infectious diseases, which, however, must be tested in randomized clinical trials. A phase I trial investigating the safety and tolerability of pembrolizumab with initial viral and immunologic outcome assessment is ongoing (NCT03239899). In addition, a pilot study has shown that nivolumab is safe and effective for the treatment of virally suppressed patients with chronic hepatitis B infection (Gane et al., 2019).

## Conclusion

Anti PD-1/PD-L1 therapies have demonstrated their promising antitumor effects in cancer immunotherapy of many different solid and hematologic malignancies. Based on the different underlying mechanism of PD-1 and PD-L1 blockade, with the evidence from real-world data, the former may be more effective than the latter, though no significant differences were found as regards to their safety profiles. However, no conclusion can be made without a randomized, head-to-head comparison between them. Future head-to-head studies are warranted for direct comparison between PD-1 and PD-L1 blockade. Moreover, with increasing understanding on the tumor microenvironment, reports to the primary and adaptive resistance to anti-PD-1/PD-L1 therapy, and *in vitro*/*ex vivo* research demonstrating the role of PD-1/PD-L1 pathway in chronic infection, there is a need to explore novel biomarkers, novel combinatorial strategies, and implement clinical trials evaluating the efficacy of PD-1/PD-L1 blockade on chronic infection, to broaden its clinical applicability in the future.
